# Toxigenic *Clostridium difficile*-mediated diarrhoea in hematopoietic stem cell transplantation in-patients: rapid diagnosis and efficient treatment

**DOI:** 10.1017/S095026882100162X

**Published:** 2021-08-10

**Authors:** Sepideh Khodaparast, Ashraf Mohabati Mobarez, Nima Khoramabadi, Mohammad Vasei, Mehdi Saberifiroozi

**Affiliations:** 1 Department of Bacteriology, Faculty of Medical Sciences, Tarbiat Modares University, Tehran, Iran; 2 Cell-based Therapies Research Centre, Digestive Disease Research Institute, Shariati Hospital, School of Medicine, Tehran University of Medical Sciences, Tehran, Iran; 3 Digestive Disease Research Institute, Tehran University of Medical Sciences, Tehran, Iran

**Keywords:** *Clostridium difficile*, hematopoietic stem cell transplant, real-time PCR

## Abstract

Allogenic hematopoietic stem cell transplant (HSCT) recipients are susceptible to any kind of infectious agents including *Clostridium difficile*. We studied 86 allogenic-HSCT patients who faced diarrhoea while receiving antibiotics. DNA from stool samples were explored for the presence of *C. difficile* toxin genes (*tcd*A; *tcd*B) by multiplex real-time PCR. Results showed nine toxigenic *C. difficile* amongst which seven were positive for both toxins and two were positive for *tcd*B. Six of toxigenic *C. difficile* organisms harbouring both toxin genes were also isolated by toxigenic culture. *Clostridium difficile* infection was controlled successfully with oral Metronidazole and Vancomycin in the confirmed infected patients.

## Introduction

Leukemic patients undergoing hematopoietic stem cell transplantation (HSCT) receive significant risk factors of graft-versus-host disease, including immunosuppressant drugs, chemotherapy and broad-spectrum antibiotics (to avoid infectious complications). Empirical antibiotic therapy in HSCT patients increases the risk of developing *Clostridium difficile* infection (CDI) during hospitalisation [1]. Considerable CDI rates of 4–27% in HSCT recipients force the early detection and control of CDI in bone marrow transplanted patients [2]. Accurate and quick detection of toxin genes in samples could be applied for patients with antibiotic-associated diarrhoea, especially those who undergo transplantation or chemotherapy. Conventionally, detection of *C. difficile* is based on a two-staged culture and PCR algorithms that confirm *C. difficile*-specific gene *glu*D (glutamate dehydrogenase (GDH); diagnosis may be completed by detecting toxins by either ELISA or tracing toxin genes with PCR-based methods. The time-consuming procedure has been suggested in guidelines as the gold standard method for approaching CDI diagnosis [3].

*Clostridium difficile* pathogenesis depends on producing toxins A and/or B (TcdA and TcdB, respectively) which are interesting targets for the detection of pathogenic types. The main objective of the present study was to evaluate the CDI rates in leukaemic patients receiving stem cell transplantation by targeting *tcd*A and *tcd*B in a rapid multiplex real-time PCR method. This procedure may be suggesting a short-cut method for considerably rapid and accurate detection of toxigenic *C. difficile*.

## Patients

From December 2017 to November 2018, 364 patients were admitted for hematopoietic stem cell transplantation. Isolation of the patients was fulfilled according to the CDC (Guideline for Isolation Precautions: Preventing Transmission of Infectious Agents in Health Care Settings) and the World Health Organisation (WHO Guidelines on Hand Hygiene in Health care). Eighty-six HSCT adult patients, receiving antibiotics and having diarrhoea (3 < unformed stools a day), were investigated for toxigenic CDIs. The Ethics Committee of Tarbiat Modares University (IR.TMU.REC.1395.403) and Digestive Disease Research Institute, Tehran University of Medical Sciences approved this survey.

Amongst 86 diarrhoeic patients, 42 had acute lymphoblastic leukaemia (ALL; 48.8%) as an underlying disease, followed by 26 chronic myelogenous leukaemia (CML; 30.2%) and 18 acute myeloid leukaemia (AML; 20.9%). According to the development of febrile periods mostly due to neutropenia, pulmonary and other relevant infections, the patients empirically received Imipenem (36%), Meropenem (24%), Ciprofloxacin (72%) and Cefixime (31%) as the most predominant antibacterial components. The history of antibiotic therapy starts from one day to one month before the diarrhoea happening.

## Multiplex real-time PCR

Stool samples were spiked with internal control of RealStar® *C. difficile* PCR Kit (altona, Germany) before extraction of total DNA by QIAamp® DNA Stool Mini Kit (Qiagen, Germany). DNA from stool samples were investigated for the presence of *tcd*A and *tcd*B by RealStar® *C. difficile* PCR Kit using both *LightCycler*^@^96 (Roche, Germany) and Rotor-gene Q (Qiagen) machines simultaneously to ensure the accuracy of real-time PCR results. Multiplex real-time PCR assessment of the stool samples showed toxigenic CDI in nine patients (10.46%) amongst which seven (8.1%) were positive for both *tcd*A and *tcd*B and two (2.32%) were only positive for *tcd*B.

## Toxigenic culture

Stool samples were collected; one spike (~1 g) of each specimen was treated by alcoholic shock for 1 h. Another one was transferred into the *Clostridium difficile* Brucella broth for 1 h under strict anaerobic conditions [4]. Treated specimens were inoculated onto the Cycloserine-Cefoxitin Fructose Agar, enriched by vitamin K1 (1 μg/ml) and hemin (5 μg/ml) and incubated in anaerobic condition for 2–5 days at 37 °C. Recovered colonies were confirmed by microbiological characteristics and the presence of *C. difficile* species-specific GDH gene (*glu*D) by PCR [5]. The presence of *tcd*A and *tcd*B genes were explored by RealStar® *C. difficile* PCR Kit as described above. Toxigenic culture yielded 10 isolates, which were confirmed as *C. difficile* by positive PCR results for *glu*D. Real-time PCR assessment of the *C. difficile* isolates for *tcd*A and *tcd*B showed six (6.97%) having both *tcd*A and *tcd*B. Four other isolated *C. difficile* were non-toxigenic.

Overall results showed that the culture method missed three toxigenic *C. difficile* and so the calculated sensitivity and specificity of the culture method are 76.92% (95% CI 46.19–94.96%) and100.00% (95% CI 95.26–100.00%), respectively. Five patients who were diagnosed with the toxigenic *C. difficile* had ALL (55.5%), two had CML (22.2%) and the other two had AML (22.2%). Pairwise two-tailed regression showed no significant correlation between the antibiotics used nor leukaemia type and the CDI in subject patients.

## Treatment

After the development of diarrhoea in patients who were receiving antibiotics, a single dose of Vancomycin and Metronidazole were administered orally (following stool sample collection) [6]. Oral treatment with Metronidazole and Vancomycin was continued for the individuals for at least 7 days upon real-time PCR confirmation of the toxigenic CDI. CDI was successfully controlled and no mortality was recorded due to the toxigenic *C. difficile* during this study.

## Discussion and conclusion

Leukaemic patients receiving allogeneic transplantation are amongst the most susceptible patient to all types of infections. Recent studies show the increasing rates of CDI from 5% to 20% and even rising higher to above 50% after the HSCT which may ruin the stem cell transplantation success [7]. Allogeneic HSCT patients are exposed to many risk factors making them susceptible to the CDI such as broad-spectrum antibiotics, chemotherapy, immunosuppression and proton pump inhibitor medications.

Our study showed that 10.4% of patients with bone marrow transplantation were infected with the toxigenic *C. difficile*; the infection rate will be increased to 15.11% if four non-toxigenic *C. difficile* isolates are included. The history of antibiotic therapy for CDI patients was from one week to one month in the post-transplantation period. We did not detect or isolate *C. difficile* from any patient who received antibiotic therapy or being hospitalised less than a week and it is compatible with the known risk of prolonged antibiotic therapy as one of the most important predisposing factors for CDI. However, we could not correlate the antibiotics used in the subject patients with the incidence of CDI.

Toxins A and B – the *C. difficile* enterotoxin and cytotoxin, respectively – are the main pathogenicity factors of the organism. Toxigenic types of *C. difficle* cause the same clinical signs of illnesses ranging from mild diarrhoea to life-threatening inflammation of colon, toxic megacolon and deadly infectious colitis [8].

Detecting *tcd*A and *tcd*B directly in fesses leads to the diagnosis of pathogenic *C. difficile* rather than targeting *glu*D gene that only confirms the presence of *C. difficile* without distinguishing between pathogen and non-pathogen types. Timing of toxigenic culture and the isolation of *C. difficile* is unfavourable when the urgency of HSCT patient cases comes into account. However, in the current study, toxigenic culture results confirm the sensitivity and specificity of the multiplex-real-time PCR detection of the pathogen.

In addition, microbiological isolation and characterisation of the toxigenic *C. difficile* may fuel further investigations such as epidemiological tracing of the pathogen transmission and the infection control measurements. Antibiotic susceptibility testing of the *C. difficile* isolates also reported to be considered [9].

Oral Metronidazole and Vancomycin were administered after collecting stool samples from HSCT patients with diarrhoea [6]. This blind treatment continued if the results approve the infection with toxigenic *C. difficile*. Multiplex real-time PCR results were obtained on the same day (fewer than four hours) and helped the precise decision on the proper use of antibiotics. Although there are widely used serological methods for screening of the *C. difficile* toxins in stool samples, real-time PCR detection of the pathogen is significantly more sensitive, specific and rapid. It remarkably improves the proper management of critically infection-susceptible HSCT patients [10].

We assume that using real-time PCR for detection of the toxigenic *C. difficile* in leukaemic/HSCT patients with antibiotic-associated diarrhoea significantly increases the accuracy of diagnosis in a short time. This leads to efficient management and control of the disease and reduces mortality due to CDI.

## Data Availability

The data that support the findings of this study are available from the corresponding author upon reasonable request.
